# Supplementary material for: Global burden and risk factors of peptic ulcer disease between 1990 and 2021: An analysis from the global burden of disease study 2021
Source: PLoS One. 2025 Jul 11;20(7):e0325821. doi: 10.1371/journal.pone.0325821 (PMC12250541; doi:10.1371/journal.pone.0325821)
Supplement: Attachment — Submitted filename: Response to Reviewers.docx [file pone.0325821.s002.docx]

Dear editor & reviewers,

Thank you for reviewing our manuscript and for the constructive comments, which greatly helped us to improve the manuscript. We have revised the manuscript in accordance with your comments. And point-by-point responses to the comments were as follows:

**Journal Requirements:**

**1.Please ensure that your manuscript meets PLOS ONE's style requirements, including those for file naming.**

Answer: Thank you for your comment and providing good suggestion for our manuscript. We have revised the manuscript based on PLOS ONE's style requirements, including those for file naming.

**2. Please note that funding information should not appear in any section or other areas of your manuscript. We will only publish funding information present in the Funding Statement section of the online submission form. Please remove any funding-related text from the manuscript.**

Answer: Thank you for your comment and providing good suggestion for our manuscript. We have removed the funding-related text from the manuscript.

**3. We note that the grant information you provided in the ‘Funding Information’ and ‘Financial Disclosure’ sections do not match. When you resubmit, please ensure that you provide the correct grant numbers for the awards you received for your study in the ‘Funding Information’ section.**

Answer: Thank you for your comment and providing good suggestion for our manuscript. We have corrected the fund information in the ‘Funding Information’ section.

**4.PLOS requires an ORCID iD for the corresponding author in Editorial Manager on papers submitted after December 6th, 2016. Please ensure that you have an ORCID iD and that it is validated in Editorial Manager. To do this, go to ‘Update my Information’ (in the upper left-hand corner of the main menu), and click on the Fetch/Validate link next to the ORCID field. This will take you to the ORCID site and allow you to create a new iD or authenticate a pre-existing iD in Editorial Manager.**

Answer: Thank you for your comment and providing good suggestion for our manuscript. We have authenticated a pre-existing iD in Editorial Manager.

**5. We note that Figure 5 in your submission contain map/satellite images which may be copyrighted. All PLOS content is published under the Creative Commons Attribution License (CC BY 4.0), which means that the manuscript, images, and Supporting Information files will be freely available online, and any third party is permitted to access, download, copy, distribute, and use these materials in any way, even commercially, with proper attribution. For these reasons, we cannot publish previously copyrighted maps or satellite images created using proprietary data, such as Google software (Google Maps, Street View, and Earth). For more information, see our copyright guidelines:** [**http://journals.plos.org/plosone/s/licenses-and-copyright**](http://journals.plos.org/plosone/s/licenses-and-copyright)**.**

Answer: Thank you for your comment and providing good suggestion for our manuscript. We have replaced Figure 6 with Tables 3 and 4.

**6. Please include captions for your Supporting Information files at the end of your manuscript, and update any in-text citations to match accordingly. Please see our Supporting Information guidelines for more information: http://journals.plos.org/plosone/s/supporting-information.**

Answer: Thank you for your comment and providing good suggestion for our manuscript. We have modified the content of the Supporting Information according to the guidelines.

**Reviewer #1:**

**(Please refer to the line numbers of the revised version of the manuscript)**

**Major issues:
1.According to the submission guidelines: Define abbreviations upon first appearance in the text. Do not use non-standard abbreviations unless they appear at least three times in the text. Keep abbreviations to a minimum.**

Answer: Thank you for your comment and providing good suggestion for our manuscript. We have deleted the unnecessary acronyms (Page7 line187, line195-196) and spelled out some of the acronyms (Page3 line66) according to the submission guidelines.

**2.Introduction: Few references in the introduction, the topic should be explored better in the literature.**

Answer: Thank you for your comment and providing good suggestion for our manuscript. We have added related references in the introduction section (Page3 line85; Page4 line90, line 93, line 96, line 100, line 103, line 106 and line 110). Meanwhile, relevant content has been added to the introduction to improve the presentation of the manuscript(Page4 line104-106 and line110; Page5 line125-126).

**3.Discussion: I had the impression that a better discussion of the results found was missing. The discussion is not a summary of the results. You should compare the results obtained with other results available in the literature and find associations that corroborate the data found.**

Answer: Thank you for your comment and providing good suggestion for our manuscript. We have added the relevant content to the discussion section. Additional comparison of study findings to prior research literature has also been provided in the discussion. (Page21 line330-332 line340-346; Page22 line352-359 line363-373; Page23 line386-389; Page24 line429-435; Page25 line441-442 line446-459)

**Minor issues:**
**Correct minor errors according to the submission guidelines:**
**1.Include page numbers and line numbers in the manuscript file. Use continuous line numbers (do not restart the numbering on each page).**

Answer: Thank you for your comment and providing good suggestion for our manuscript. We have used page numbers and line numbers in the manuscript file.

**2.Figure captions are inserted immediately after the first paragraph in which the figure is cited. Figure files are uploaded separately. Use figure label with Arabic numerals, and “Figure” abbreviated to “Fig” (e.g. Fig 1, Fig 2, Fig 3, etc).**

Answer: Thank you for your comment and providing good suggestion for our manuscript. The figure captions were inserted immediately after the first paragraph in which the figures were cited, and the figure labels were also modified according to the guidelines. The figure files were also uploaded separately.

**3.Materials and methods
Add the website as a reference instead of placing it in the middle of the text (https://vizhub.healthdata.org/gbd-results/).**

Answer: Thank you for your comment and providing good suggestion for our manuscript. We have replaced the website with reference 22 in our manuscript (Page5 line141).

**4.Discussion: Please find a more recent reference to replace reference number 19.**
Answer: Thank you for your comment and providing good suggestion for our manuscript. We have found a more recent reference to replace reference number 19(Page21 line326).
**5."which indicated that the PUD burden was significantly heavier in males, potentially due to lower levels of estrogen" - This assumption needs a reference or better argumentation.**
Answer: Thank you for your comment and providing good suggestion for our manuscript. We have added the relevant content and corresponding references accordingly(Page21 line340-345).
**6."which appeared to be associated with limited access to diagnostic services." - A reference is needed for this assumption.**

Answer: Thank you for your comment and providing good suggestion for our manuscript. We have added recent references for the above sentence(Page23 line401).

**Reviewer #2:**

**(Please refer to the line numbers of the revised version of the manuscript)**

**Q0: A similar study using the same material was published in 2023: "Peptic ulcer disease burden, trends, and inequalities in 204 countries and territories, 1990–2019: a population-based study" ( doi: 10.1177/17562848231210375). Beyond sex (male patients being more vulnerable in terms of incidence, prevalence mortality and DALY) and smoking (a primary risk factor), were there any other notable findings comparing the 2023 study to the current one? Additionally, did the COVID-19 pandemic influence the epidemiological characteristics of peptic ulcer disease between 2020 and 2021? If not, what are the authors' perspectives on this.**

Answer: Thank you for your questions. The latest version of the 2021 dataset was released on the official website of the Global Burden of Disease (GBD) public database on May 16, 2024. To investigate the latest epidemiological situation of Peptic Ulcer Disease (PUD), we utilized the most recent data from 2021 for our analysis. Coupled with the literature you referenced, our study presents the following primary innovations:

1.We analyzed the numbers and age-standardized incidence, prevalence, deaths and DALYs rates of peptic ulcer disease in 2021, whereas the literature analyzed the numbers and age-standardized incidence rates in 2019.

2. We analyzed the age-specific numbers and rates of PUD globally in 2021, whereas the literature did not include related research.

3.We analyzed the trends in the all-age numbers and age-standardized incidence, prevalence, mortality and DALYs rates of PUD by sex from 1990 to 2021, whereas the literature did not include related research.

4. We did the Joinpoint regression analysis of the sex-specific ASRs for PUD globally from 1990 to 2021, whereas the literature did not include related research.

5. We analyzed the age-standardized rates of PUD globally and for 21 GBD regions by SDI from 1990 to 2021, and also analyzed the age-standardized rates of PUD in 204 countries globally by SDI in 2021, whereas the literature analyzed the numbers and age-standardized incidence rates by SDI in 2019.

6. We employed the ARIMA model to predict trends in Age-standardized rates of PUD for both sexes globally over the next 29 years (2022-2050), whereas the literature did not include related research.

7. We analyzed the risk factors for PUD based on the GBD database, whereas the literature did not include related research.

Answer: I think that the COVID-19 pandemic may affect the epidemiological characteristics of PUD between 2020 and 2021. The specific impacts can be analyzed from the following aspects:

1.Redistribution of Medical Resources and Delayed Diagnosis
The COVID-19 pandemic led to a global redistribution of medical resources, particularly during the peak of the pandemic, when many hospitals shifted their focus to treating COVID-19 patients. This resulted in delays or interruptions in the diagnosis and treatment of many routine diseases, including peptic ulcers. Many patients may not have sought medical attention in time, leading to delayed diagnosis and treatment, which in turn affected ulcer incidence and mortality.

2.Helicobacter pylori Transmission and Treatment Interventions
Helicobacter pylori is one of the primary causative factors of peptic ulcers. The COVID-19 pandemic strengthened public health measures, including social distancing and hygiene protocols, which may have influenced the transmission rate of H. pylori. Meanwhile, the pandemic caused treatment delays, and patients might have missed routine H. pylori eradication therapy, potentially worsening peptic ulcers.

3.Changes in Stress and Unhealthy Lifestyle Habits
During the COVID-19 pandemic, there was unprecedented psychological and social stress worldwide. Many people faced unemployment, isolation, and health crises, significantly increasing stress levels. Stress is a key trigger for peptic ulcers, and prolonged anxiety and psychological pressure could exacerbate ulcer development. Additionally, lifestyle changes during the pandemic, such as irregular eating habits, lack of exercise, smoking, and alcohol abuse, may have contributed to the onset and progression of peptic ulcers.

4.Impact of Non-Steroidal Anti-Inflammatory Drug (NSAID) Use
During the COVID-19 pandemic, some patients may have misused non-steroidal anti-inflammatory drugs (NSAIDs) for symptoms such as pain or fever, which could have worsened the occurrence of peptic ulcers. Since NSAIDs are known gastrointestinal ulcer risk factors, particularly with long-term use, this medication pattern during the pandemic may have led to an increased incidence of ulcers.

5.Reduction in Public Health Policies and Medical Screening
At the beginning of the pandemic, many countries restricted health screenings and treatments for non-acute diseases, which may have resulted in delayed diagnosis and treatment for peptic ulcer patients, particularly in areas severely affected by the pandemic. These changes may have influenced the statistical data for PUD in 2020 and 2021, especially in terms of early diagnosis and screening.

In conclusion, the COVID-19 pandemic has had multiple impacts on the epidemiological characteristics of many kinds of diseases, including PUD. Although we observed a potential decrease in ulcer incidence in some regions, factors such as strained medical resources, increased psychological stress, unhealthy lifestyle habits, and treatment delays may have posed challenges to the onset and treatment of peptic ulcers. The combined effect of these factors may have led to delayed diagnosis during the pandemic, and even increased the severity of the disease. Therefore, it is essential to maintain a focus on the prevention, diagnosis, and treatment of peptic ulcers in the post-pandemic context.

**Q1: The authors noted in the Discussion section that 'the ASRs of PUD remain abnormally high in Taiwan.' However, in the 2023 study mentioned in Q0 stated that 'EAPCs of ASDR were significantly decreasing in ... Taiwan'. What are the authors' opinions on this?**

Answer: Thank you for your question. In our study, we found that Taiwan (Province of China) (335.31 (95% UI: 284.75 to 399.55) per 100,000) had the highest age-standardized prevalence rate (ASPR) in 2021, whereas the literature showed that the EAPCs of age-standardized DALYs rates (ASDR) were significantly decreasing in Taiwan (Province of China) from 1990 to 2019. Futhermore, the EAPC (Estimated Annual Percentage Change) is used to measure the trend of changes in a specific disease indicator (such as incidence rate, mortality rate, or disability-adjusted life years) over a defined period. The above analyses are different both in terms of analytical perspectives and time periods.

**Q2: The ASRs of incidence, prevalence, deaths and DALYs of PUD decrease more rapidly in males than in females. Please discuss the possible reasons for this phenomenon.**

Answer: Thank you for your comment and providing good suggestion for our manuscript. We have added the corresponding content in the discussion section. [Smoking](https://www.geenmedical.com/article?id=36685897&type=true) and alcohol consumption have been recognized as risk factors for PUD. Previous research has shown that the prevalence of active smoking among men decreased from 60.0% to 47.6%, while the proportion of daily alcohol consumption declined from 19.0% to 13.1% between 1997 and 2020, which could partly explain the aforementioned decreasing trends for males(Page22 line355-359).

**Q3: How can the causal relationship between peptic ulcer disease (PUD)-related disability-adjusted life years (DALYs) and cigarette smoking be determined?**

Answer: Thank you for your question. The Population Attributable Fraction (PAF) is the proportion of the outcome that would be removed if a risk factor had been reduced to the theoretical minimum risk exposure level (TMREL). The GBD 2021 utilized the comparative risk assessment framework to estimate DALYs and deaths for PUD attributable to specific risk factors.

PAF Calculation:

PAF=P(RR-1)/P(RR-1) + 1

P represents the prevalence of smoking in the population, and RR is the relative risk of smoking associated with PUD. The GBD attribution analysis model is used to estimate the contribution of smoking to PUD-related DALYs across different populations.

**Q4: In "Global and regional burden of PUD", the authors mentioned: "The Taiwan (Province of China) (335.31 (95% UI: 284.75 to 399.55) per 100,000) had the highest ASPR." Though this annotation '(Province of China)' was shown in IHME forms, Taiwan is not globally recognized as a province of China; if it were, China would not pursue its annexation. For instance, does China plan to annex Hebei or Guangdong (both Province of China) in the future? Please remove the annotation '(Province of China)' to avoid potential political issues in scientific research.**

Answer: Thank you for your comment and providing good suggestion for our manuscript. We have removed the annotation '(Province of China)' in the manuscript(Page18 line259, Page23 line395).

**Reviewer #3:**

**(Please refer to the line numbers of the revised version of the manuscript)**

**1. The introduction, while providing necessary background, suffers from a lack of specific focus and a strong "hook" to immediately engage the reader. It lacks a clearly stated hypothesis and a concise explanation of the study's novel contributions to the existing literature. Furthermore, the objectives are stated too broadly. To improve, provide a brief "roadmap" of the paper's structure.**

Answer: Thank you for your comment and providing good suggestion for our manuscript. The latest version of the 2021 dataset was released on the official website of the Global Burden of Disease (GBD) public database on May 16, 2024. Recent studies on Peptic Ulcer Disease (PUD) using the GBD database were based on 2019 data and had a relatively limited research scope. To investigate the latest epidemiological situation of PUD, we utilized the most recent data from 2021 for our analysis. We have revised our manuscript to try to address these issues in the introduction section. We believe this revision has enhanced the clarity and focus of the manuscript, providing a clearer explanation of the study's significance(Page4 line104-106 line110, Page5 line125-126).

The structure of this paper is outlined as follows, comprising five main sections:

Section1：

**Numbers and ASRs of PUD in different regions in 1990 and 2021**

**Table2**

**Table1**

Section2：

**Numbers and ASRs of PUD globally by age and sex**

**Fig1**

**Fig2=2**

**Fig3**

Section3：

**ASRs of PUD by SDI or in the world map**

**Table3**

**Fig5=2**

**Fig4**

**Table4**

Section4：

**Predicted trends in ASRs of PUD globally in 2022-2050**

**Fig6=2**

Section5：

**Risk factor estimation for PUD**

**Fig7=2**

References for analyses similar to our paper are as follows:

(1) Dong XJ, Wang BB, Hou FF, Jiao Y, Li HW, Lv SP, Li FH. Global burden of atrial fibrillation/atrial flutter and its attributable risk factors from 1990 to 2019. Europace. 2023 Mar 30;25(3):793-803. doi: 10.1093/europace/euac237.

(2) GBD 2017 Oesophageal Cancer Collaborators. The global, regional, and national burden of oesophageal cancer and its attributable risk factors in 195 countries and territories, 1990-2017: a systematic analysis for the Global Burden of Disease Study 2017. Lancet Gastroenterol Hepatol. 2020 Jun;5(6):582-597. doi: 10.1016/S2468-1253(20)30007-8.

**2. The manuscript uses an** **ARIMA model to predict PUD burden until 2050. Long-term epidemiological predictions are inherently unreliable due to numerous unpredictable factors (e.g., changes in healthcare policy, emergence of new treatments, shifts in risk factor prevalence, and unforeseen global events). The manuscript fails to adequately acknowledge and address these limitations.**

Answer: Thank you for your comment and providing good suggestion for our manuscript. You are absolutely correct that long-term epidemiological predictions are inherently subject to uncertainties due to various unpredictable factors, including changes in healthcare policy, the emergence of new treatments, shifts in the prevalence of risk factors, and unforeseen global events. While the ARIMA model provides a useful framework for making projections based on historical data and we have found some similar literature(1. Zhang X, Zhao L, He M, Huang X, Liu D. Burden of silicosis based on the Global Burden of Disease Study 2021: trend analysis of incidence, mortality, and disability-adjusted life years, and projections for the next 30 years. J Thorac Dis. 2025 Feb 28;17(2):872-886. doi: 10.21037/jtd-24-1341. 2. Ma X, Liu R, Xi X, Zhuo H, Gu Y. Global burden of chronic kidney disease due to diabetes mellitus, 1990-2021, and projections to 2050. Front Endocrinol (Lausanne). 2025 Feb 21;16:1513008. doi: 10.3389/fendo.2025.1513008. 3. Jena D, Padhi BK. Global and regional burden of cardiovascular diseases due to non-optimal temperature exposure: a systematic analysis with forecasts to 2050. QJM. 2025 Jan 7:hcaf002. doi: 10.1093/qjmed/hcaf002), we acknowledge that these predictions should be interpreted with caution. In the revised manuscript, we will include a more thorough discussion of the limitations inherent in long-term predictions, emphasizing that such projections should be viewed as potential scenarios rather than definitive outcomes. Additionally, we will highlight the need for periodic updates to the model as new data and evidence emerge to assess how different assumptions might affect the results. Thank you again for your insightful comment, and we will incorporate these considerations to improve the clarity and robustness of the manuscript(Page24 line429-435).

**3.The conclusion that smoking is the only primary risk factor associated with DALYs and deaths from PUD is an overstatement not supported by a comprehensive analysis. The authors present this conclusion without exploring potential confounding factors or interactions between different risk factors.**

Answer: We greatly appreciate your insightful comment regarding our conclusion that smoking is the only primary risk factor associated with DALYs and deaths from PUD. We acknowledge that this statement may appear to be an overstatement without a thorough exploration of potential confounding factors, and are happy to provide a detailed explanation on this matter. When conducting the GBD analysis, the selection of risk factors is based on the data and availability provided by the GBD database. In the 2021 and earlier versions of the GBD database, the risk factors for PUD did not cover all known potential factors but focused on analyzing key factors that influence the burden of PUD based on global epidemiological evidence and data sources. In the current GBD data, smoking is identified as the primary modifiable risk factor associated with PUD and has therefore been treated as a central factor in our analysis. However, as you rightly pointed out, Helicobacter pylori infection, long-term use of non-steroidal anti-inflammatory drugs (NSAIDs), and other factors are clearly important risk factors for PUD, with varying impacts across regions and populations. Due to differences in the datasets of the GBD database by region or time period, some datasets may lack certain factors, resulting in only smoking being included as an independent risk factor for PUD. In the revised version, we will further clarify the data limitations and suggest that a more comprehensive analysis could be conducted as new data becomes available(Page3 line73; Page24 line417 line437; Page25 line441-442 line452-456).

**4. The manuscript reports gender differences in PUD burden but fails to provide a meaningful explanation for these disparities. Simply stating that males have higher rates than females is insufficient. The authors do not explore the underlying biological, behavioral, or socioeconomic factors that could explain these differences.**

Answer: Thank you for your comment and providing good suggestion for our manuscript. We have added relevant content in the discussion part and appropriately cited the sources to provide a meaningful explanation for these disparities (Page21 line340-345).

**5. The conclusion states that "precise prevention strategies are essential to mitigate the disease burden of PUD," but this statement is generic and lacks specific, actionable recommendations. The manuscript does not provide a clear link between the study's findings and concrete policy implications. The authors do not discuss how the observed trends and risk factors should inform the development and implementation of targeted prevention programs.**

Answer: We appreciate the feedback and agree that the conclusion would benefit from more specific and actionable recommendations. In response, we have revised the conclusion to better align the study's findings with concrete policy implications (Page25 line446-451; Page25 line454-459). We hope these revisions meet the expectations and provide a clearer link between our findings and actionable prevention programs.

**6. Finally, while the language is generally understandable, the manuscript contains several minor grammatical and stylistic errors that detract from its overall clarity and professionalism. A thorough proofreading by a native English speaker is recommended to address these issues and ensure the** **manuscript meets the standards for publication.**

Answer: We appreciate your valuable feedback regarding the language and clarity of the manuscript. We acknowledge the presence of minor grammatical and stylistic errors and have taken steps to address them. A thorough proofreading has been conducted to ensure the manuscript meets the highest standards of clarity and professionalism. We believe these revisions improve the overall readability of the manuscript. Thank you for your helpful suggestion!
