# Supplementary material for: Global burden and risk factors of peptic ulcer disease between 1990 and 2021: An analysis from the global burden of disease study 2021
Source: PLoS One. 2025 Jul 11;20(7):e0325821. doi: 10.1371/journal.pone.0325821 (PMC12250541; doi:10.1371/journal.pone.0325821)
Supplement: Attachment — Submitted filename: Response to academic editor and reviewer(s)..docx [file pone.0325821.s004.docx]

Dear academic editor,

Thank you for reviewing our manuscript and for the constructive comments, which greatly helped us to improve the manuscript. We have revised the manuscript in accordance with your comments. And point-by-point responses to the comments were as follows:

**1. Incomplete Correction Regarding “Taiwan (Province of China)”**

**Despite the authors’ indication that all instances of 'Province of China' were removed from references to Taiwan, at least one instance remains in the main manuscript. We appreciate that such an error may have been unintentional. However, given this is the second or third time the issue is recurring, the sensitivity of the terminology and prior revision instructions, it is essential that all instances are carefully corrected in the next revision. Please comprehensively review the entire manuscript, including main text, figure captions, tables, and supplementary materials, to ensure full removal of ‘Province of China’ references, consistently referring to the location simply as ‘Taiwan’ as recommended by the reviewer.**

Answer: We sincerely thank you for pointing out the remaining reference to “Province of China” in relation to Taiwan. We apologize for this oversight and fully acknowledge the importance and sensitivity of this terminology.

In the revised manuscript with track changes, we have conducted a comprehensive review of the entire manuscript, including the main text, figures, figure captions, tables, and supplementary materials. We have carefully removed all instances of “Province of China” and have consistently used “Taiwan” throughout the document, in accordance with the reviewer’s recommendation and the journal’s guidance.

The reference to "Province of China" has been removed both in the main text and in Figure 5, as shown below:

page 19, line 258:

page 20, line 272-273:

page 25, line 406:

Figure 5:

We greatly appreciate your thorough review and your guidance, which have been instrumental in improving the quality and clarity of our manuscript.

Sincerely,
Huachong Ma
On behalf of all co-authors

**2.** **Methodological Concern: ARIMA Model Projections**

**Authors use the ARIMA model to project the future burden of peptic ulcer disease up to 2050. However, some projections—notably age-standardised mortality rates (ASMR) and disability-adjusted life-years (ASDR) for females—predict negative rates by 2050. Negative incidence or mortality rates are biologically implausible. Authors need to revise the ARIMA modelling approach to either constrain the lower limit of projections to zero or explain the biological and methodological limitations of ARIMA predictions in the Discussion section and avoid interpreting negative projections literally.**

Answer: We appreciate your insightful observation regarding the ARIMA-based projections, specifically the biologically implausible negative values in the age-standardised mortality rate (ASMR) and disability-adjusted life years (ASDR) for females by 2050. We fully agree that negative incidence or mortality rates are not biologically plausible. The ARIMA model, while widely used for time-series forecasting, is purely statistical in nature and does not incorporate biological constraints. As such, it may yield unrealistic values—particularly when the input data demonstrate long-term declining trends or when projections are extended far into the future, as in our case. To address the concern regarding the appearance of implausible (e.g., negative values in the age-standardised rates) in the long-term forecast, we have revised our analysis by shortening the ARIMA model-based prediction period to 2022–2040. This adjustment ensures the stability and interpretability of the results while remaining aligned with the available historical trend data. The relevant S1 file, figure (figure6) and descriptions (page 3, line 80; page 5, line 139; page 7, line 206; page 21, line 277-294; page 25, line 415-416; page 39, line 888-897; page 40, line 909) have been modified and uploaded accordingly.

We thank you again for pointing out this important issue and believe these changes substantially improve the rigor and clarity of our analysis.

Sincerely,
Huachong Ma
On behalf of all co-authors

**3. Language and Grammar**

**Although the manuscript is generally understandable, several grammatical errors remain, including but not limited to: typographical errors, incorrect article usage, and redundant wording. A full, careful language edit (sentence-by-sentence) is necessary to ensure clarity and professional presentation. Authors may seek assistance from a professional editing service.**

Answer: Thank you for your valuable feedback regarding the language quality of our manuscript. In response, we have carefully revised the entire manuscript with close attention to grammar, article usage, typographical errors, and overall clarity. A thorough sentence-by-sentence edit has been conducted to enhance the professionalism and readability of the text. We believe the revised version now meets the journal’s language standards.

Specifically, we made the following changes:

**Abstract:** Corrected word usage and reworded several sentences to improve clarity and eliminate awkward phrasing (page 2, line 44-48, line 56-58; page 3, line 81-82, line 86-87).

**Introduction:** Corrected word usage to improve clarity (page 5, line 141).

**Methods:** Corrected word usage to improve clarity (page 5, line 151-152, line 157).

**Results:** Enhanced the precision of expressions and corrected word usage to improve clarity and eliminate awkward phrasing (page 8, line 245-248, line 252-253; page 16, line 179-182, line 184-185, line 188-191; page 17, line 201; page 18, line 224, line 237-238).

**Discussion and Conclusion:** Enhanced the precision of expressions and corrected word usage to improve clarity and eliminate awkward phrasing (page 24, line 390; page 25, line 402-403, line 408, line 420; page 27, line 466).

We hope that the revised version is now acceptable in terms of language quality. Please let us know if further revisions are needed.

Sincerely,
Huachong Ma
On behalf of all co-authors

**Journal Requirements:**

**Please review your reference list to ensure that it is complete and correct. If you have cited papers that have been retracted, please include the rationale for doing so in the manuscript text, or remove these references and replace them with relevant current references. Any changes to the reference list should be mentioned in the rebuttal letter that accompanies your revised manuscript. If you need to cite a retracted article, indicate the article’s retracted status in the References list and also include a citation and full reference for the retraction notice.**

Answer: Thank you for your kind reminder regarding the reference list. In response, we have conducted a thorough review of all cited references to ensure their completeness, accuracy, and current validity.

We have made the following adjustments:

**1.Replacement of improperly formatted or duplicate references:**

(1) Reference [5] was found to be formatted incorrectly (e.g., missing journal issue numbers, inconsistent citation styles). New reference [5]: Shen HY, Li X, Ye BX, Jiang JX. A fundic gland polyp and peptic ulcer in Meckel's diverticulum. Dig Liver Dis. 2022 Oct;54(10):1439-1440. https://doi.org/10.1016/j.dld.2022.02.004 PMID: 35236642→ The change has been marked in the revised manuscript on Page29, line606-608.

(2) We identified two instances of duplicate references in the manuscript (previous references [6] and [9] which both cited the same article by Laucirica I et al., 2023). Therefore reference [9] has been removed, and a more relevant, recent article has been cited in its place: Kavitt RT, Lipowska AM, Anyane-Yeboa A, Gralnek IM. Diagnosis and Treatment of Peptic Ulcer Disease. Am J Med. 2019 Apr;132(4):447-456. https://doi.org/10.1016/j.amjmed.2018.12.009 PMID: 30611829 → The change has been marked in the revised manuscript on Page29, line620-622.

(3) We identified two instances of duplicate references in the manuscript (previous references [8] and [10] which both cited the same article by Hudnall A et al., 2022). Therefore reference [10] has been removed, and a more relevant, recent article has been cited in its place: Markar SR, Vidal-Diez A, Patel K, Maynard W, Tukanova K, Murray A, et al. Comparison of Surgical Intervention and Mortality for Seven Surgical Emergencies in England and the United States. Ann Surg. 2019 Nov;270(5):806-812. https://doi.org/10.1097/SLA.0000000000003518 PMID: 31567504→ The change has been marked in the revised manuscript on Page29, line623-626; Page30, line637.

**2. Addition of DOI and PMID for All References:**

We have revised the entire reference list to include both DOI and PMID (where available) for every cited publication. This enhances the traceability and accessibility of cited sources. All updates have been applied to the reference section in the revised manuscript and are reflected in the tracked changes version → The change has been marked in the revised manuscript on Page28-39.

**3. Reference Style and Formatting:**

Minor inconsistencies in punctuation, author listing, journal abbreviations, and publication dates have been corrected to meet the journal’s style guidelines → The change has been marked in the revised manuscript on Page28-39.

All reference-related revisions have been clearly noted in the main text and are summarized in the rebuttal letter accompanying the revised manuscript. We believe these updates enhance the academic rigor and transparency of our submission.

Thank you again for your guidance and the opportunity to improve our manuscript.

Sincerely,
Huachong Ma

On behalf of all authors
