# Supplementary material for: Global burden and risk factors of peptic ulcer disease between 1990 and 2021: An analysis from the global burden of disease study 2021
Source: PLoS One. 2025 Jul 11;20(7):e0325821. doi: 10.1371/journal.pone.0325821 (PMC12250541; doi:10.1371/journal.pone.0325821)
Supplement: Attachment — Submitted filename: Response to academic editor.docx [file pone.0325821.s005.docx]

Dear academic editor,

Thank you for reviewing our manuscript and for the constructive comments, which greatly helped us to improve the manuscript. We have revised the manuscript in accordance with your comments. And point-by-point responses to the comments were as follows:

**ACADEMIC EDITOR:** Thank you for your revised submission. The manuscript is greatly improved, and your responses have addressed the reviewers’ and editor’s concerns. However, a few minor issues remain that require correction before the manuscript can be accepted.

**1. First, peptic ulcer disease (PUD) is described as a ‘prevalent acute abdominal condition’, which could be misleading. PUD is a chronic gastrointestinal condition that may present acutely when complications arise. Please revise this description for accuracy or provide justification for using ‘acute’.**

Answer: Thank you for your valuable feedback regarding the description of peptic ulcer disease (PUD). We agree that referring to PUD as a “prevalent acute abdominal condition” may lead to misunderstanding, given its chronic nature. Accordingly, we have revised the manuscript to describe PUD as a chronic gastrointestinal condition that can present acutely when complications occur. This revision clarifies that while PUD itself is chronic, acute presentations often necessitate urgent clinical attention.

The revised sentence now reads:
“Peptic ulcer disease (PUD) is a chronic gastrointestinal disorder that may present acutely due to complications and poses significant clinical and economic challenges.” (page 2, line 42-44)

We believe this change accurately reflects the clinical characteristics of PUD and aligns with current medical understanding.

**2. Second, there are minor discrepancies between the structured abstract and the full abstract, including a spelling error (‘identifed’ should be ‘identified’) and some formatting inconsistencies.**

Answer: Thank you for pointing out the discrepancies between the structured abstract and the full abstract. We have carefully reviewed both sections to ensure consistency in content, structure, and formatting. Specifically, the typographical error “identifed” has been corrected to “identified” (page 3, line 85), and the expressions were refined and word usage corrected to enhance clarity and eliminate awkward phrasing. (page 3, line 85-86, line 88)

We appreciate your attention to detail and believe these corrections enhance the clarity and presentation of the manuscript.

**3.Finally, please take this opportunity to review the manuscript sentence by sentence to ensure accuracy, clarity, and consistency throughout.**

Answer: We sincerely appreciate your recommendation. In response, we have conducted a thorough, sentence-by-sentence revision of the entire manuscript to enhance its linguistic accuracy, clarity, and consistency. Specifically, we:

- Refined grammatical structures and improved word choices throughout the revised manuscript.
- Eliminated redundant or awkward phrasing to ensure smooth and professional expression.
- Corrected all identified typographical and formatting inconsistencies, including in the structured and unstructured abstracts.
- Ensured consistency in terminology and abbreviations.

We hope these efforts meet the expectations of the editorial team and enhance the overall readability and quality of the manuscript.
